# Construction of a novel signature and prediction of the immune landscape in gastric cancer based on necroptosis-related genes

**DOI:** 10.1038/s41598-022-15854-8

**Published:** 2022-08-02

**Authors:** Zhengtian Li, Wenkang Yang, Dejun Liu, Weizheng Ye, Gang Du, Xi Li

**Affiliations:** 1 Department of Bone and Joint Surgery, The First Affiliated Hospital of Guangxi Medical University, Nanning, 530021 Guangxi China; 2 Guangxi Medical University, Nanning, Guangxi China; 3 The First Affiliated Hospital of Guangzhou University of Chinese Medicine, Guangzhou, Guangdong China

**Keywords:** Gastric cancer, Liver cancer, Pancreatic cancer, Genetics, Immunology, Microbiology

## Abstract

Necroptosis, a type of programmed cell death, has become a potential therapeutic target for solid tumors. Nevertheless, the potential roles of necroptosis-related genes (NRGs) in gastric cancer (GC) remain unknown. The objective of the present study was to create a necroptosis-related prognostic signature that can provide more accurate assessment of prognosis in GC. Using The Cancer Genome Atlas (TCGA) and Gene Expression Omnibus (GEO) data, we identified differentially expressed NRGs. Univariate analysis and Lasso regression were performed to determine the prognostic signature. Risk scores were calculated and all GC patients were divided into high- and low-risk score group according to the median risk score value. The robustness of this signature was externally validated with data from GSE84437 cohort (n = 431). Survival analysis revealed high-risk score patients had a worse prognosis. Results evidenced that the signature was an independent prognosis factor for survival. Single-sample sequence set enrichment analysis (ssGSEA) exhibited different enrichment of immune cells and immune-related pathways in the two risk groups. Furthermore, a predictive nomogram was generated and showed excellent predictive performance based on discrimination and calibration. In addition, the risk score positively correlated with tumor mutational burden and was associated with sensitivity to multiple anti-cancer drugs. Overall, our work demonstrates a close relationship between necroptosis and the prognosis of GC. The signature we constructed with potential clinical application value, can be used for prognosis prediction and being a potential therapeutic responses indicator in GC patients.

## Introduction

Gastric cancer (GC) is a leading contributor to global cancer mortality^1^. Attributed to the lack of early and effective detection methods, GC with early metastasis is characterized by poor prognosis, unsatisfactory treatment effect^2,3^. To date, surgical resection is currently the therapy of choice for the most of patients^4^. For most early stages of GC patients, clinical symptoms are atypical and the signs are not obvious, causing delayed diagnosis, missed diagnosis and loss of chance for surgical excision. Therefore, this raises an urgent need for developing more effective diagnostic, prognostic and therapeutic biomarkers.

As early as 2005, Alexei discovered a programmed cell death pattern that is different from normal cell apoptosis: necroptosis, and then be confirmed as a unique pattern of cell death^5^. Unlike other programmed apoptosis, necroptosis is a form of regulated cell death that is characterized by formation of the necrosome complex based on swelling injury^6^. Necroptosis may accelerate cancer cell death or enhance the sensitivity of tumor cells to anti-cancer treatment^7–10^. Research has demonstrated that necroptosis is able to overcome resistance to cancer drugs mediated by P-glycoprotein, Bcl-2, and Bcl-xL in cancer cell lines^11^. Zhao et al. found that necroptosis-related lncRNAs could predict prognosis and help make a distinction between the cold and hot tumors for improving individual therapy in GC^12^. All these results indicated necroptosis may be a potent therapeutic target for the treatment of cancer. Nevertheless, the precise role of necroptosis-related genes (NRGs) in GC still not clear. Hence, understanding the impact of NRGs on GC development may provide potential prognostic biomarkers and therapeutic targets and guide immunotherapy strategies for GC.

In this study, we aimed to develop a necroptosis-related prognostic signature (NRGsig) with guiding significance for GC prognosis and immunotherapy. We successfully divided GC patients into two necroptosis-related molecular subtypes with diverse clinical outcomes and tumor microenvironment (TME) infiltration characteristics. Furthermore, we created the NRGsig to quantify the level of necroptosis and assist prognosis assessment and therapeutic decision making for individual patients with GC.

## Materials and methods

### Data collection and processing

We first mined the Cancer Genome Atlas (TCGA) database (https://portal.gdc.cancer.gov/) to obtain all the raw data, including mutation data, copy number variation (CNV) information, RNA-sequencing (FPKM) and clinical data of 375 GC samples and 32 normal samples. Gene expression dataset of GSE84437 were retrieved from the GEO database (https://www.ncbi.nlm.nih.gov/geo/) and included 431 GC samples. Patients with incomplete clinical data were excluded. The clinical characteristics of GC samples from TCGA-GC and GSE84437 cohorts were presented in Table 1. To eliminate batch effects of different cohorts, we converted the fragments per kilobase million (FPKM) values into Transcripts Per Million (TPM) values by data.table, tibble, dplyr, and tidyr R packages. After data correction, the transcriptome RNA sequences of the TCGA-GC and GSE84437 cohorts were merged as meta-cohort by using the “ComBat” algorithm of the “SVA” package.

**Table 1:** Clinicopathologic characteristics of gastric cancer patients in TCGA and GSE84437 cohorts.

Variables	TCGA-GC cohort	GSE84437 cohort
	(n = 375)	(n = 431)
	N (%)	N (%)
Age (M ± SD, years)	65.22 ± 10.51	60.02 ± 11.58
**Age**		
≤ 60	125 (33.3)	194 (28.3)
> 60	250 (66.7)	237 (71.7)
**Gender**		
Female	135 (36.0)	137 (45.5)
Male	240 (64.0)	294 (54.5)
**Grade**		
G1-2	140 (37.3)	–
G3	235 (62.7)	–
**Stage**		
I	47 (12.5)	–
II	122 (32.5)	–
III	172 (45.9)	–
IV	34 (9.1)	–

### Identification of differentially expressed NRGs and mutation analysis

By mining the Gene Set Enrichment Analysis (GSEA) (http://www.gsea-msigdb.org/gsea/index.jsp), we acquired the necroptosis gene set M24779.gmt, which contains eight necroptosis-associated genes. After an extensive literature search about necroptosis, we ultimately identified 67 NRGs^13–15^ (details are presented in Table S1). Differentially expressed NRGs (DENRGs) in tumor and normal tissues in the TCGA-GC cohort were screened using the “limma” package, with *p* < 0.05. Using the “maftools” package, we depicted the somatic mutation plots of DENRGs in GC patients. The plots of CNV alterations and the chromosomal location for DENRGs were generated using the“Circos” package. DENRGs were uploaded in STRING database (https://string-db.org/cgi/input.pl) and then performed the protein–protein interaction (PPI) networks using cytoscape (version 3.7.2). Kyoto Encyclopedia of Genes and Genomes (KEGG) pathway^16–18^ and Gene ontology (GO) term analysis were also performed to those DENRGs using "clusterProfiler", "org.Hs.eg.db", "enrichplot", and "ggplot2" R packages.

### Consensus clustering analysis

To identify different molecular subtypes related with necroptosis, We conducted consensus clustering using the “ConsensusClusterPlus” package based on the expression of DENRGs^19^, and then cycled 1000 times to ensure accurate and stable clustering. Kaplan–Meier (KM) curves were performed using the “survival”and“survminer”packages. In addition, the TME infiltration characteristics among molecular subtypes were performed using CIBERSORT algorithm. *P* < 0.05 were considered significant.

### Differences analysis among molecular subtypes

Differentially expressed genes (DEGs) between distinct molecular subtypes were screened using the “limma” package, with adjusted P < 0.05 and |Log2(fold change) |> 1^20^. Enrichment analysis based on these DEGs was also performed via the “clusterProfiler” R package^21^. Adjusted *P* < 0.05 was adopted as significant.

### Construction and validation of necroptosis-related prognostic signature

Prognostic associated genes were screened from DEGs by univariate Cox regression analysis and were next subjected to the lasso Cox regression in TCGA-GC cohort and yielded optimal genes^22–24^. Thus, a prognostic signature that calculates individual risk scores was established and we termed as NRGsig. The formula patients’ risk scores was described below: Risk score = ∑Expgenei*βi, where Expgene represents the relative expression value of the optimal genes, and β represents the regression coefficient. Kaplan–Meier curves and receiver operating characteristics (ROC) curves were performed to assess the sensitivity and specificity of NRGsig. In addition, investigation was performed using principal component analysis (PCA) and T-distributed neighbor embedding (T-SNE) to analyze whether the prognostic model might properly categorize patients into two risk groups^25^. In addition, the GSE84437 cohort was used as an external validation set to confirm the model’s predictive value. Patients with GC were stratified into distinct groups based on age (≤ 60 or > 60 years), sex (female or male), grade (G1-2 and G3), T-stage (T1-2 and T3-4), N-stage (N0 and N1-3), and M-stage (M0 and M1). To explore the impact of the NRGsig on the clinicopathological features of GC, the R package “survminer” was applied to investigate the correlation between risk score and the above clinical features.

### Development of a prognostic nomogram and comparison of the prognostic signatures

Univariate Cox analysis and multivariate Cox analysis were performed to evaluate the prognostic value of clinical factors including risk score. We generated a nomogram integrating independent prognostic factors as a convenient tool for the prediction of 1, 3, and 5-year OS in individual GC patients using the “rms” package. The discrimination, accuracy, and practicability of the nomogram were evaluated using the ROC curve, calibration curve (with 1000 bootstrap resamples), and decision curve analysis (DCA)^26^, respectively. To compare efficacy between the NRGsig and other multigenetic signatures of previous GC studies^27–30^, time-dependent ROC curve, C-index and Kaplan–Meier curve analysis were carried out.

### GSEA enrichment analysis and comparison of immune activity among subgroups

GSEA was adopted to analysis the difference of functions and pathway between high and low risk group. Analysis of single-sample sequence set enrichment (ssGSEA) was utilized using the “gsva” package^31,32^. The enrichment score of immune cells and immune-related activities in two groups was explored in TCGA-GC and GSE84437 cohort. Tumor mutation burden (TMB) score for each GC patient in TCGA-GC cohort was calculated via Perl scripts. Spearman correlation analysis were performed to investigate the correlation of TMB and risk score and the TMB score in different risk groups was performed and performed. In addition, different expression of immune checkpoint genes between high- and low-risk score groups was investigated.

### Evaluation of the chemotherapy drugs and immunotherapy response

The half inhibitory concentration (IC50) of chemotherapeutic and targeted therapeutic drugs in different risk groups were calculated by the “pRRophetic” R package^33,34^, then we predicted the drugs sensitivity between two risk groups to chemotherapy^35–37^. Immunophenoscore (IPS) of the TCGA-GC cohort was downloaded from The Cancer Immunome Atlas (TCIA; https://tcia.at/home; Table S2). PD1 and CTLA4 were the candidate immune checkpoints enrolled for IPS analysis. Groups with higher IPS scores will benefit more from immunotherapy^38^. Potential immunotherapeutic responses were also predicted with Tumor Immune Dysfunction and Exclusion (TIDE, Table S3) algorithm in the TCGA-GC cohort based on the transcriptome profiles. A low TIDE score represents a good response towards immunotherapy^39^.

### Ethics approval and consent to participate

TCGA and GEO belong to public datasets. The patients involved in the databases have obtained ethical approval. Users can download relevant data for free for research and publish relevant articles. This manuscript is not a clinical trial; hence, ethics approval and consent to participate is not applicable. All the procedures were performed in accordance with the relevant guidelines and regulations.

## Results

### The landscape of genetic variation of DENRGs in GC

**Figure 1:**
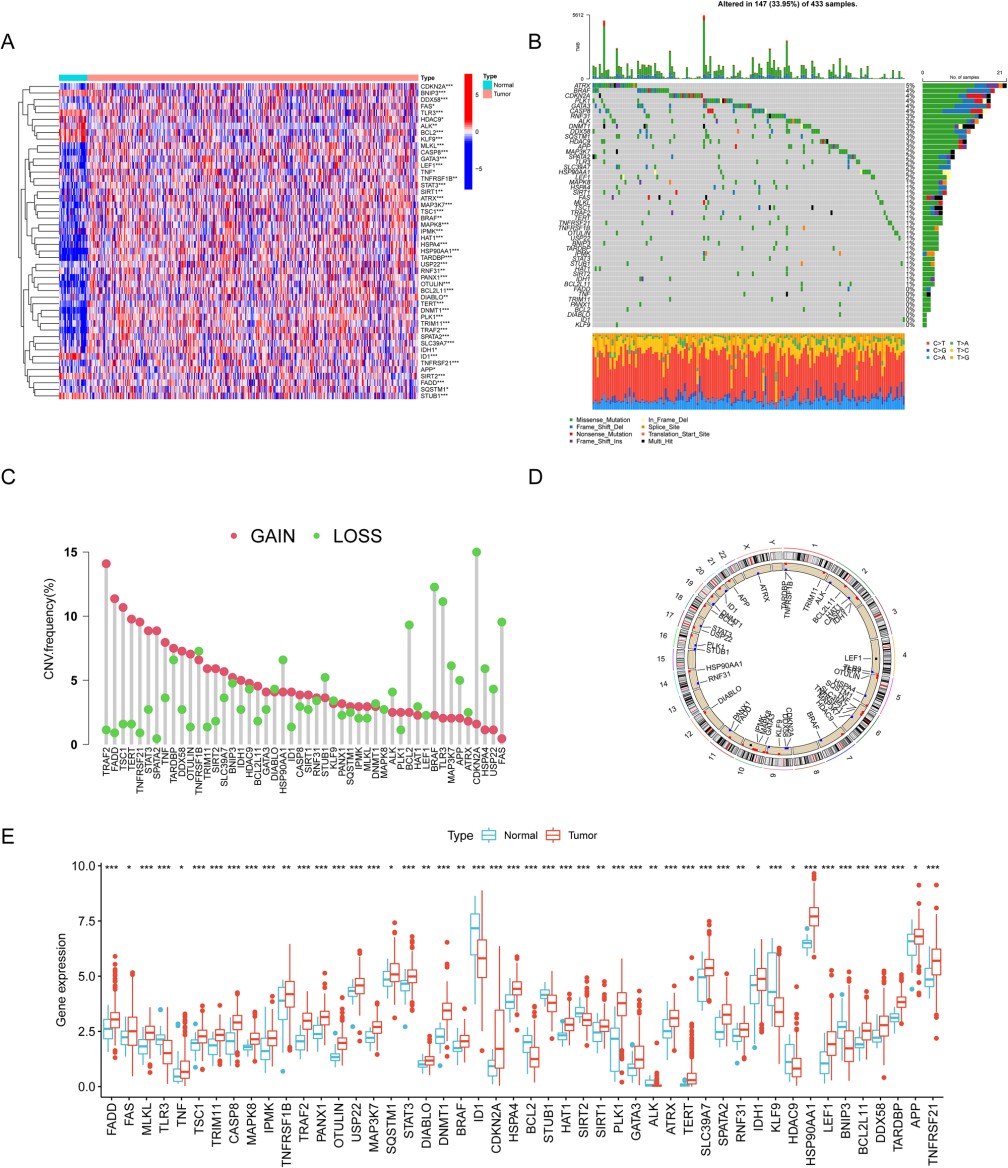
The landscape of genetic alterations of DENRGs in GC. (**A**) Heatmap of DENRGs expression between the normal and tumor samples. Blue represents normal gastric tissue, pink represents tumor tissue; upregulated genes were defined as red, and downregulated genes as blue. (**B**) Mutation characteristics of DENRGs in the TCGA-GC cohort. The TMB is presented in the barplot at the top of the image; the mutation frequency of each DENRGs is indicated on the barplot right. The barplot on the right represents different mutation types proportions. (**C**) CNV variants frequency of the DENRGs in the TCGA-GC cohort. Red: amplification frequency. Green: loss frequency. The column represented the alteration frequency. (**D**) The locations of CNV alteration of DENRGs on 23 chromosomes. (**E**) Expression of DENRGs between normal gastric tissue and tumor tissue. Blue: normal gastric tissue. Red: tumor tissue. DENRGs, differentially expressed necroptosis-related genes. (*p < 0.05; **p < 0.01; ***p < 0.001).

A total of 48 DENRGs were identified using “limma” package for further analysis (*p* < 0.05, Fig. 1A). Out of 433 GC samples, 147 (33.95%) were showed regulatory mutations associated with necroptosis (Fig. 1B) and ATRX (5%) was the highest frequency mutated gene**.** As loss or gain of function is commonly achieved through DNA mutation or amplification/deletion, we considered both somatic mutation and somatic copy number changes in our analysis. We first summarized the incidence of copy number variations and somatic mutations of 48 DENRGs in GC. The frequency of CNV alterations and found that all 48 DENRGs showed prevalent CNV alterations (Fig. 1C). The rates of amplification or deletion for most of DENRGs were relatively low. The altered position of CNVs of DENRGs on chromosome were also scanned and illustrated with visual figure (Fig. 1D). In addition, most of the DENRGs were significant increase in tumor tissues (Fig. 1E).

To further explore the interactions of these DENRGs, we conducted a PPI analysis, and the PPI network was shown in Fig. S1A. In addition, the correlation network containing all DENRGs was presented in Fig. S1B. The network above indicated that there was a very strong correlation among DENRGs. GO-term analysis showed that DENRGs were associated with necrotic cell death, programmed necrotic cell death, necroptotic process and apoptotic signaling pathway (Fig. S2A). KEGG pathway analysis displayed that these DENRGs were involved in multiple tumor-related signaling pathway including necroptosis, apoptosis, TNF signaling pathway, IL-17 signaling pathway, and Toll-like receptor signaling pathway (Fig. S2B).

### Identification of necroptosis subtypes in GC

**Figure 2:**
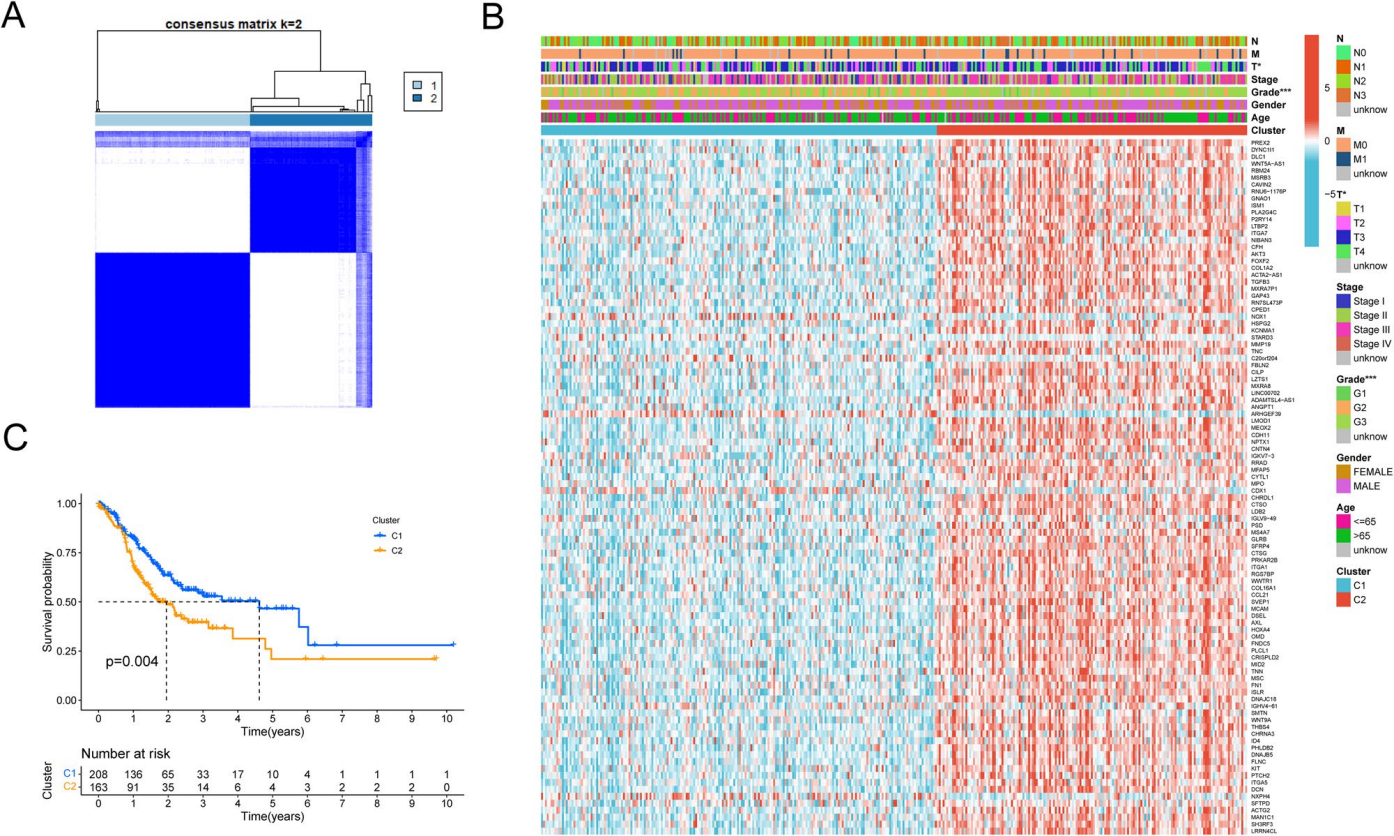
Tumor molecular subtypes related by differentially expressed necroptosis-related genes. (**A**) Consensus clustering of GC patients for k = 2 in the meta-cohort (TCGA-GC and GSE84437). (**B**) Unsupervised clustering heatmap of top 100 DEGs in GC. Clusters, age, gender, grade and stage were used as patient annotations. Red represents high DEGs expression and blue low DEGs expression. *p < 0.05; **p < 0.01; ***p < 0.001. (**C**) Kaplan–Meier curves (Log-rank test, *P* = 0.004) for OS of two necroptosis-related molecular subtypes. Blue line represents cluster C1 (n = 208), yellow line represents cluster C2 (n = 163). DEGs, differentially expressed genes between various molecular subtypes; OS, overall survival.

According to Consensus clustering analysis, when the clustering variable was set to the optimal value (K = 2), the intragroup correlations were the highest, and the intergroup correlations were the lowest, indicating that all GC patients could be classified into two molecular subtypes (Figs. 2A, S3A and S3B), which were termed as C1 (n = 208) and C2 (n = 163). The heatmap demonstrated a significant difference between cluster C1 and C2 in clinical factors including tumor grade and T stage (Fig. 2B). Result of Kaplan–Meier curve analysis revealed that the patients in C2 cluster had a poorer prognosis (Fig. 2C). The results above indicated that the necroptosis subtypes classified by consensus clustering analysis do well in distinguishing prognosis of those GC patients.

**Figure 3:**
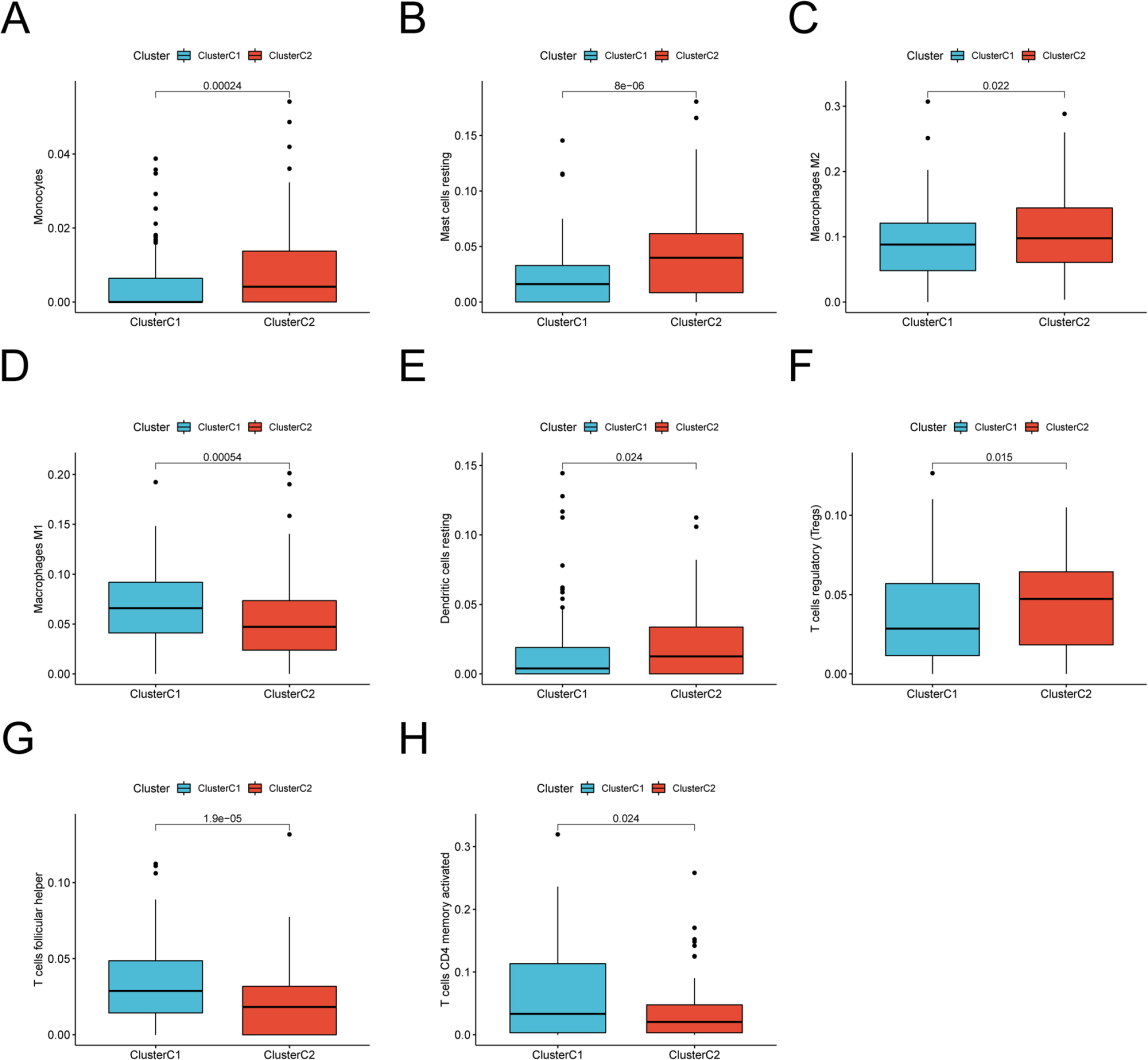
TME immune cell infiltration levels between two molecular subtypes. The abundance of Monocytes (**A**), resting Mast cells (**B**), M2 macrophages (**C**), M1 macrophages (**D**), resting Dendritic cells (**E**), T cells regulatory (Tregs) (**F**), T cells follicular helper (**G**) and activated T cells CD4 memory (**H**) between the two subtypes (all *p* < 0.05). Blue represents cluster C1, red represents cluster C2. The median value is represented as the thick line, and the interquartile range is represented as the box bottom and top. Scattered dots represent outliers.

Given the clear importance of the TME in tumorigenesis, we further investigated whether the two subtypes showed differential characteristics of immune microenvironment and the main results presented in Fig. 3A–H. The abundance of immune infiltrating cells, including resting Dendritic cells, resting Mast cells, T cells regulatory (Tregs), Monocytes and M2 macrophages, were found significantly higher in the C2 subtype. And M1 macrophages, T cells follicular helper and activated T cells CD4 memory in C1 subtypes showed greater infiltration. These results suggested that the two molecular subtypes associated with necroptosis had distinct TME infiltration characteristics and prognoses.

### Identification of DEGs associated with necroptosis phenotype

**Figure 4:**
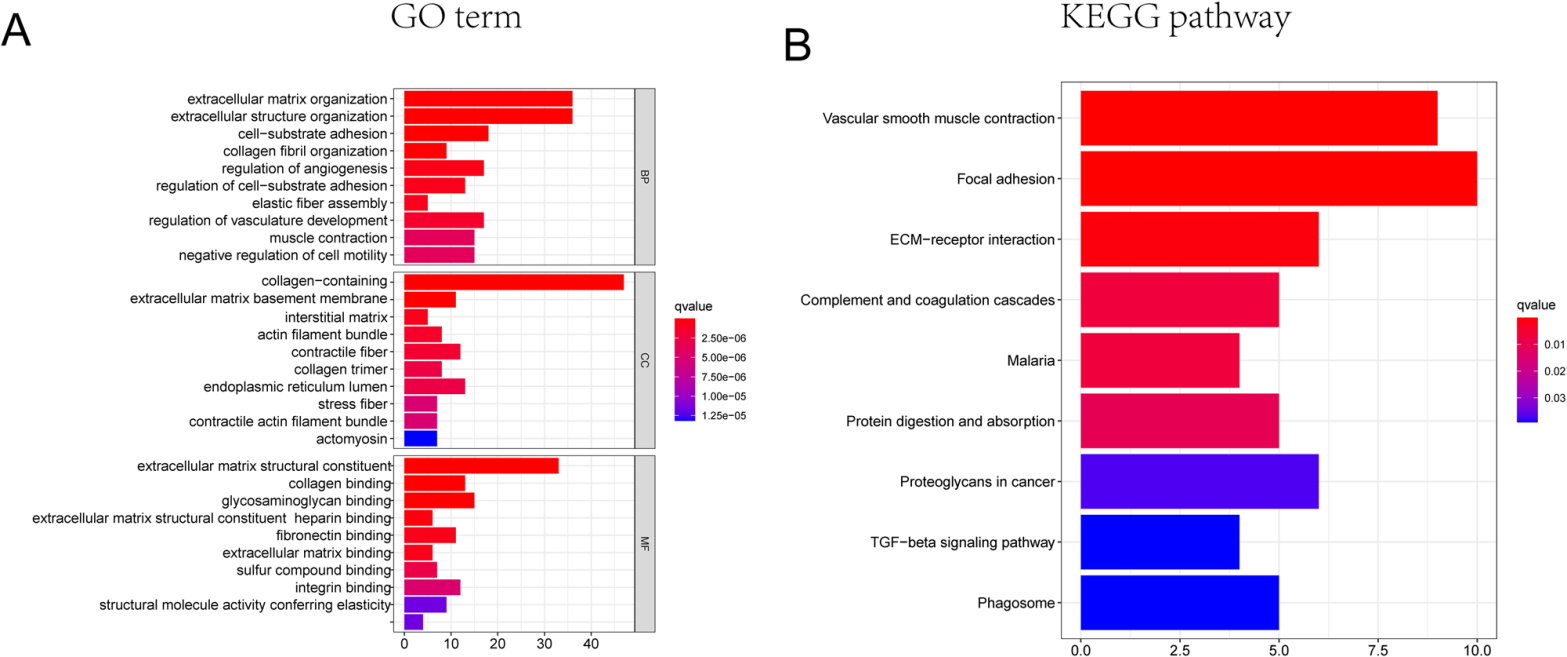
Functional enrichment analysis of the DEGs. (**A**) Top 10 enriched GO terms of the DEGs (**B**) Top 10 enriched KEGG pathways of the DEGs. The box color represents the number of enriched genes. Red represents a large number of genes enriched; blue is the opposite. *DEGs* differentially expressed genes, *BP* biological process, *CC* cellular component, *MF* molecular function. (all adjusted *p* < 0.05).

To better understand the mechanisms responsible for the prognosis differences in the two above molecular subtypes, we further investigate the functional and pathway and 1101 DEGs associated with necroptosis phenotypes were identified by the “limma” package. GO analysis showed an enrichment of GO terms for these DEGs, including extracellular matrix organization, collagen containing and extracellular matrix binding (Fig. 4A). KEGG pathway analysis for the DEGs showed that genes involved in immune-related pathways were enriched, including ECM-receptor interaction, Focal adhesion, and TGF-beta signaling pathway (Fig. 4B). These results reconfirmed a pivotal role of necroptosis in regulating the immune microenvironment.

### Construction and validation of NRGsig based on necroptosis-related subtypes

**Figure 5:**
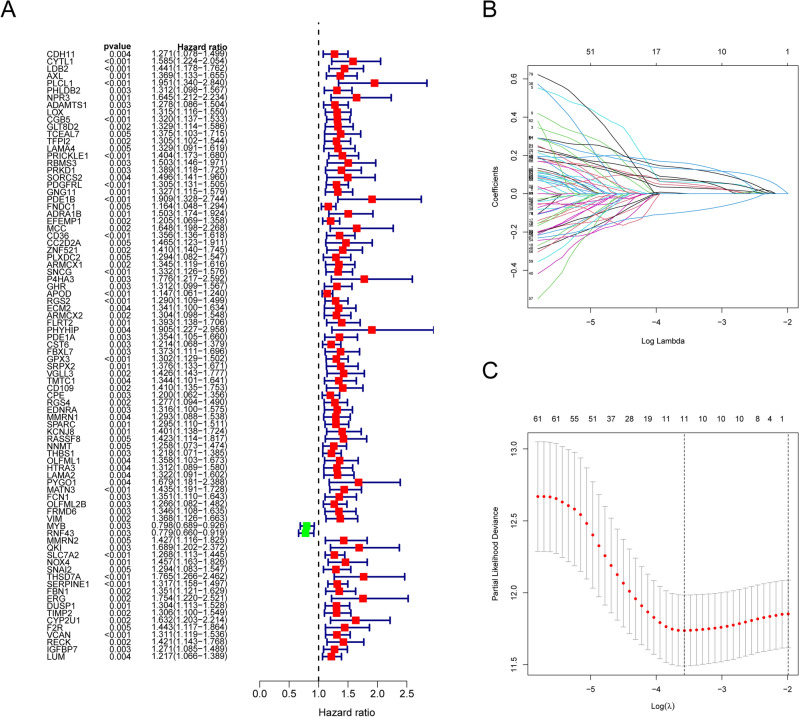
The development of NRGsig in the TCGA-GC cohort. (**A**) The prognostic-related genes determined by univariate Cox-regression analysis. Red represents risk genes; green represents protective genes. (**B**) LASSO regression of prognostic-related genes. (**C**) Cross‐validation for tuning the parameter selection.

Although our results identify a role of necroptosis molecular subtypes in prognosis and regulation of immune infiltration, these analyses are based only on patient groups and cannot be used to predict the necroptosis characteristics in individual GC patients. For this, we next constructed an multigenic prognostic signature associated with prognosis and response to treatment in each GC patient based on differential genes of molecular subtypes. We performed univariate Cox regression analysis on all DEGs and resulted in 84 genes as candidate genes (all *P* < 0.005; Fig. 5A). Most of the candidate genes were risk factors for the prognosis of GC except for MYB and RNF43. We then subjected the candidate genes to LASSO Cox regression analysis by narrowing the number of genes for the establishment of the NRGsig (Fig. 5B and C). In total, 11 optimal genes (CYTL1, PLCL1, CGB5, ADRA1B, APOD, RGS2, CST6, MATN3, RNF43, SLC7A2 and SERPINE1) were screened (Table 2) and most of the optimal genes were significant differential expression between the normal tissue and tumor tissue (Fig. S4). The formula of the risk score was calculated as follow:

$$\begin{gathered} Risk\; score = CYTL1 {\text{exp}}.\; \times \;0.05351 \; + \; PLCL1 {\text{exp}}.\; \times \;0.06101 \; + \; CGB5 {\text{exp}}.\; \times \;0.1605 \hfill \\ \quad \quad \quad \quad \, + \; ADRA1B {\text{exp}}.\; \times \;0.07886\; + \;APOD {\text{exp}}.\; \times \;0.03166\; + \;RGS2 {\text{exp}}.\; \times \,0.04199\, + \;CST6 {\text{exp}}. \hfill \\ \quad \quad \quad \quad \, \times \;0.00119 \; + \;MATN3 {\text{exp}}.\; \times \;0.13379 \; + \;RNF43 {\text{exp}}.\; \times \; - 0.09577\; + \, SLC7A2 {\text{exp}}. \times \;0.07123. \hfill \\ \quad \quad \quad \quad \, + \;SERPINE1 {\text{exp}}.\; \times \;0.12925 \hfill \\ \end{gathered}$$

**Table 2:** Correlation coefficients of 11 optimal genes in necroptosis-related prognostic signature.

Gene	Coefficient	Type
CYTL1	0.05351	Up regulated
PLCL1	0.06101	Up regulated
CGB5	0.16050	Up regulated
ADRA1B	0.07886	Up regulated
APOD	0.03166	Up regulated
RGS2	0.04199	Up regulated
CST6	0.00119	Up regulated
MATN3	0.13379	Up regulated
RNF43	-0.09577	Down regulated
SLC7A2	0.07123	Up regulated
SERPINE1	0.12925	Up regulated

**Figure 6:**
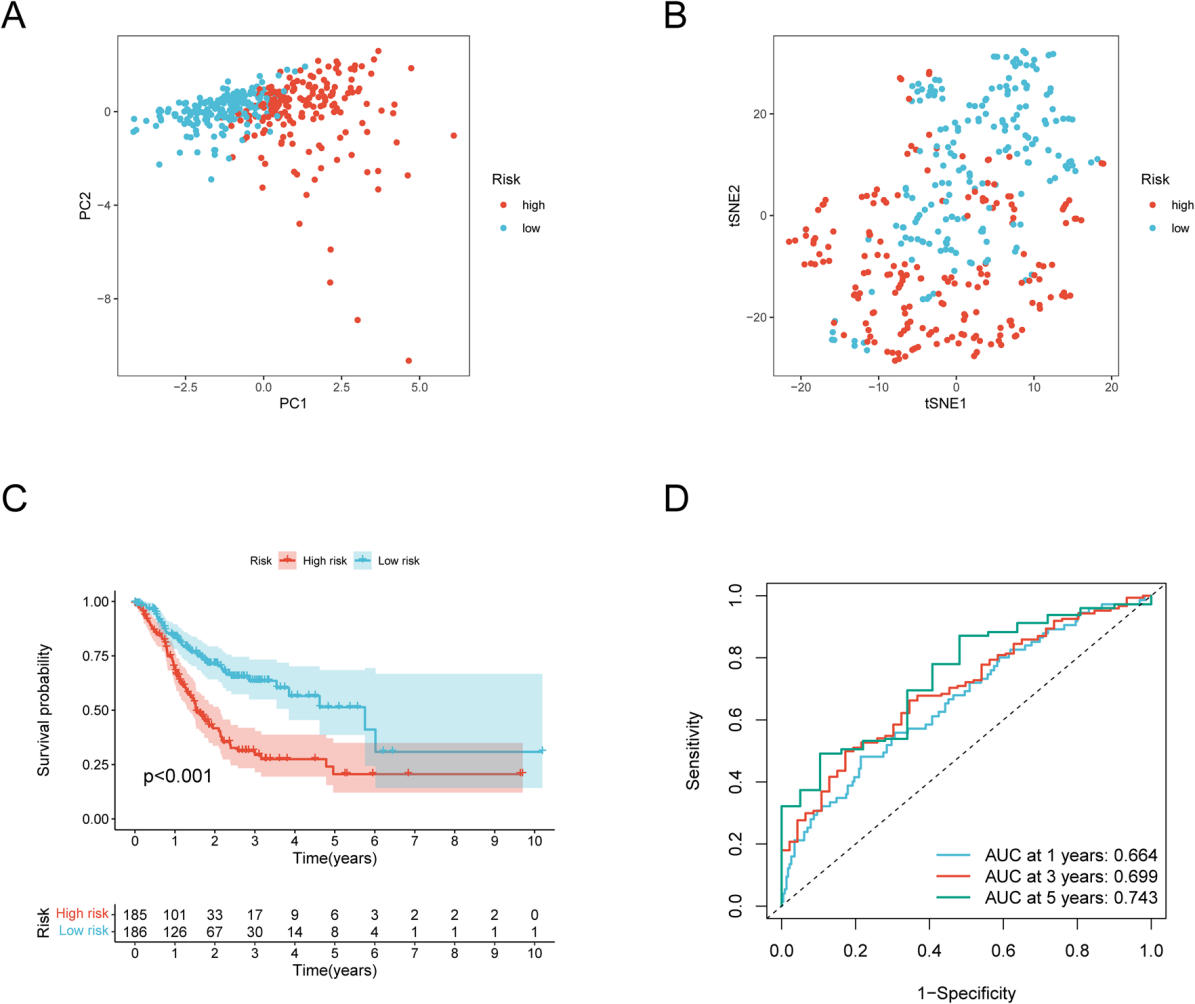
Prognosis value of necroptosis-related prognostic signature in the TCGA-GC cohort. (**A**) Principal component analysis plot. (**B**) T-distributed neighbor embedding plot. (**C**) Kaplan–Meier curves (Log-rank test, *P* < 0.001) for OS of high- and low-risk groups. (**D**) The AUC of the prediction of 1, 3, 5‐year survival rate of GC. OS, overall survival.

All GC patients were divided into high- and low-risk score group according to the median risk score value. Next, we investigated whether the prognostic signature could distinguish different risk groups of patients clearly. A clearly discernable dimensions between the two risk groups of patients was observed according to the results of PCA and t-SNE analysis (Fig. 6A and B). Kaplan–Meier curves analysis revealed high-risk group patients had a worse prognosis. (Fig. 6C). The time-dependent ROC curves were performed to evaluate the prediction performance of the NRGsig and the areas under the curve for 5-year was 0.743 in the TCGA-GC cohort (Fig. 6D). Results above demonstrated NRGsig’s advantage as robust tool for prognosis.

### Validation of the NRGsig

**Figure 7:**
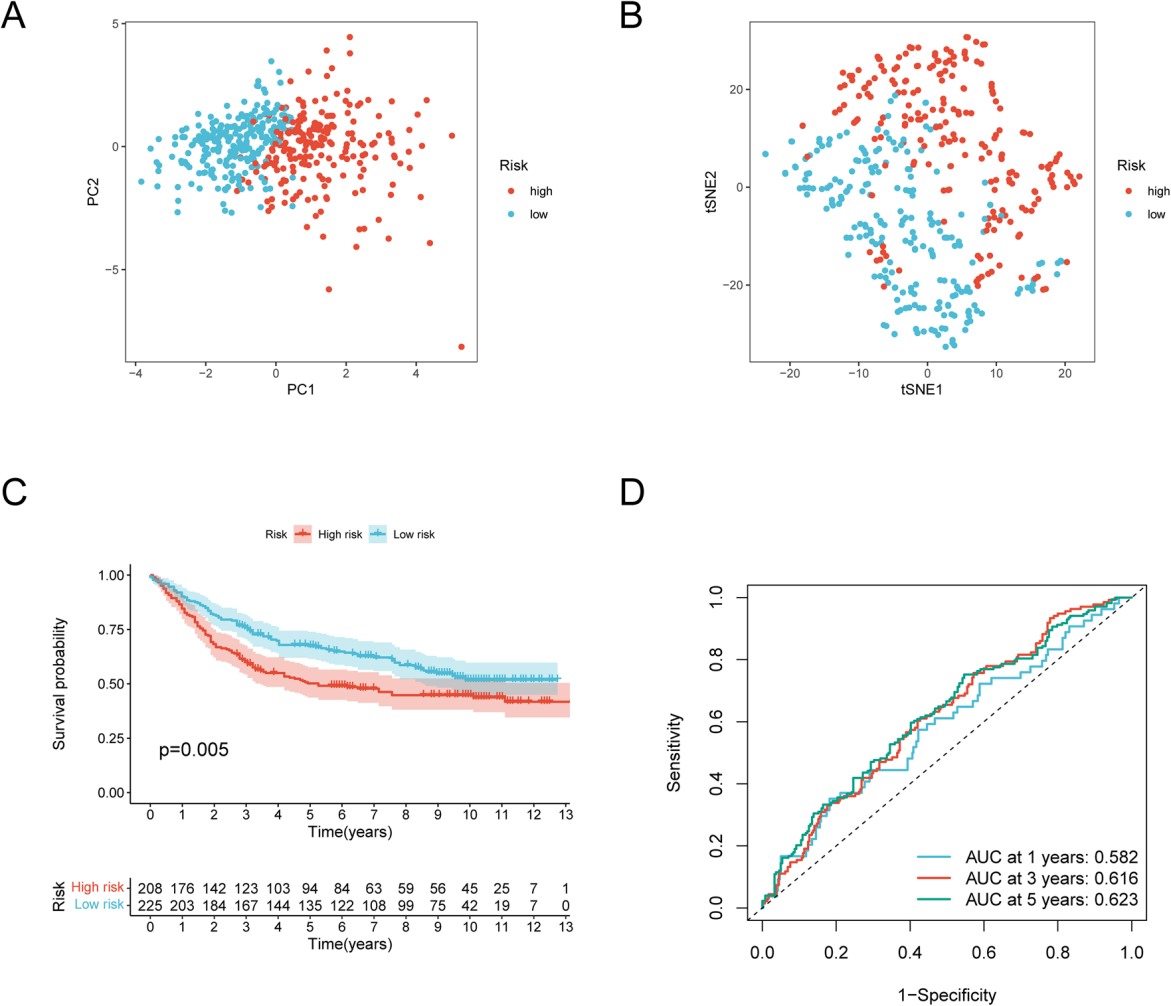
Validation of the necroptosis-related prognostic signature in the GSE84437 cohort. (**A**) Principal component analysis plot. (**B**) T-distributed neighbor embedding plot. (**C**) Kaplan–Meier curves (Log-rank test, *P* = 0.005) for OS of high- and low-risk groups. (**D**) The AUC of the prediction of 1, 3, 5‐year survival rate of GC. OS, overall survival.

We externally validated the NRGsig using the GSE84437 dataset, an independent validation dataset, and found a similar prediction performance. Patients were then classified as being high or low risk according to the calculated NRGsig risk score. A clearly two directions between the two risk groups of patients was also observed according to the results of PCA and t-SNE analysis (Fig. 7A and B). Kaplan–Meier curves analysis indicated high-risk group patients had a worse outcome (Fig. 7C). This independent validation dataset yielded a prediction performance AUC of 0.623 at 5-year (Fig. 7D). As a whole, these results showed a satisfactory prediction performance of the NRGsig in external data.

### Independent prognostic value of the NRGsig

**Figure 8:**
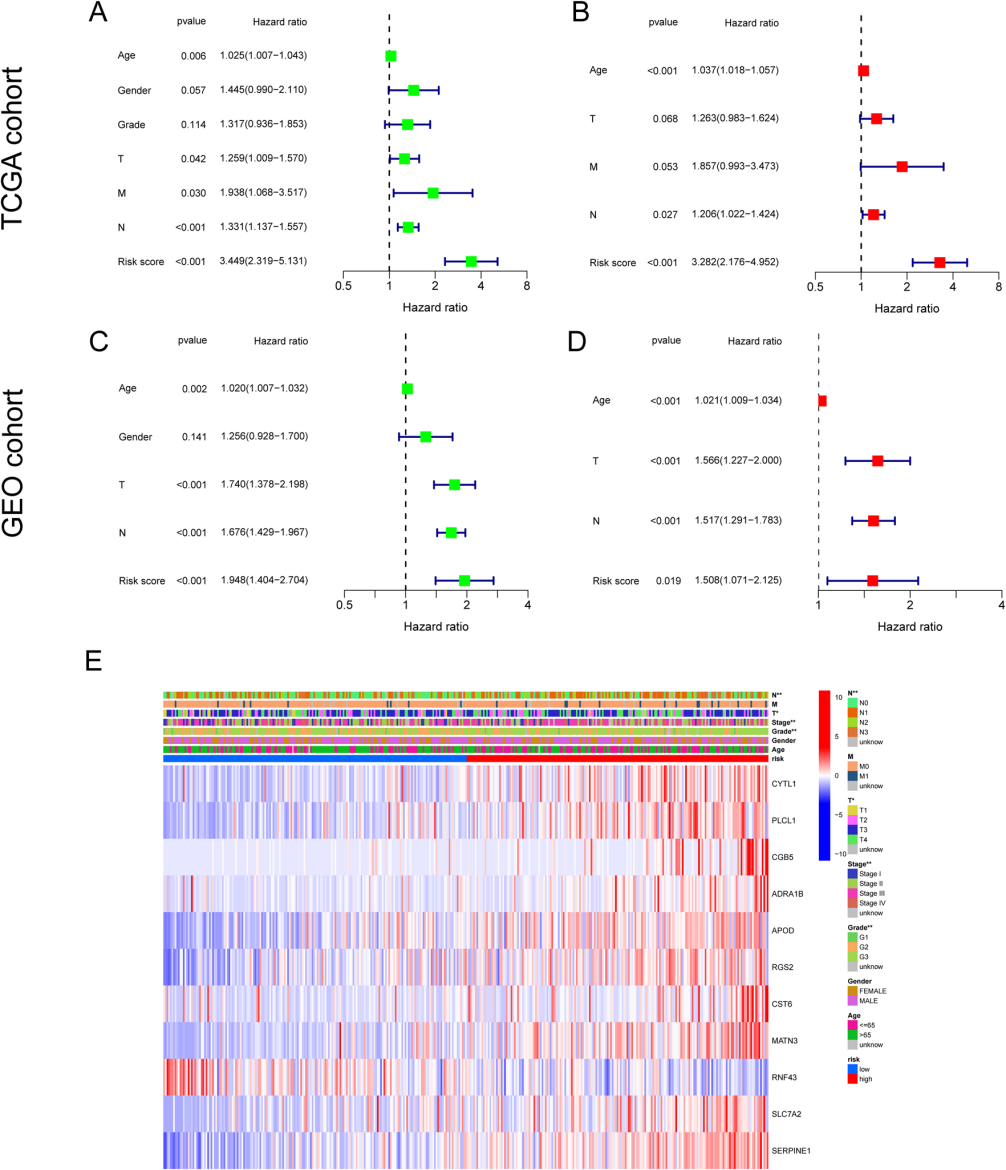
Independent prognosis analysis. (**A**, **B**) Univariate Cox regression analysis in the TCGA-GC cohort. (**C**, **D**) Multivariate Cox regression analysis in the GSE84437 cohort. (**E**) Heatmap depicting the clinicopathological characteristics and optimal genes expression between the high- and low-risk groups. Risk, age, gender, grade and stage were used as patient annotations. Red represents high expression and blue low expression. *p < 0.05; **p < 0.01; ***p < 0.001.

The independence of NRGsig were evaluated by univariate and multivariate Cox regression analysis and the result revealed the NRGsig was an independent prognostic factor of GC (Fig. 8A and B). Above analysis were repeated in the GSE84437 cohort and similar results were observed (Fig. 8C and D). Furthermore, the clinical features in the different risk groups for TCGA-GC cohort we depicted as a heatmap (Fig. 8E). To verify the clinical implications of our NRGsig risk score, we examined the correlation of the risk score with the available clinical features in TCGA-GC cohort. The Kaplan–Meier curves indicated that risk score remained its independent predictive performance regardless of other clinical features, including age (≤ 60 or > 60 years), sex (female or male), grade (G1-2 and G3), T-stage (T3-4), N-stage (N0 and N1-3), and M-stage (M0) (Fig. S5A–L). Survival analysis demonstrated that these 11 optimal genes were all correlation with the OS of GC patients (Fig. S6A–K). All the results above illustrated that NRGsig was a satisfactory and reliable prognostic tool and could be as an independent risk factor for GC.

### Gene set enrichment analysis

After categorizing cases of TCGA-GC cohort into two risk score groups by the median risk score value, we further performed GSEA analysis towards them. The results of GSEA suggested that the KEGG_COMPLEMENT_AND_COAGULATION_CASCADES, KEGG_ECM_RECEPTOR_INTERACTION, KEGG_FOCAL_ADHESION, KEGG_HYPERTROPHIC_CARDIOMYOPATHY_HCM, and KEGG_NEUROACTIVE_LIGAND_RECEPTOR_INTERACTION were the top five most enriched pathways in the high-risk group, while the KEGG_CELL_CYCLE, KEGG_DNA_REPLICATION, KEGG_BASE_ EXCISION_REPAIR, KEGG_RIBOSOME, and KEGG_SPLICEOSOME pathways were most enriched in the low-risk group (Figs. S7A and B).

### Construction of the nomogram and comparison of the prognostic signature

**Figure 9:**
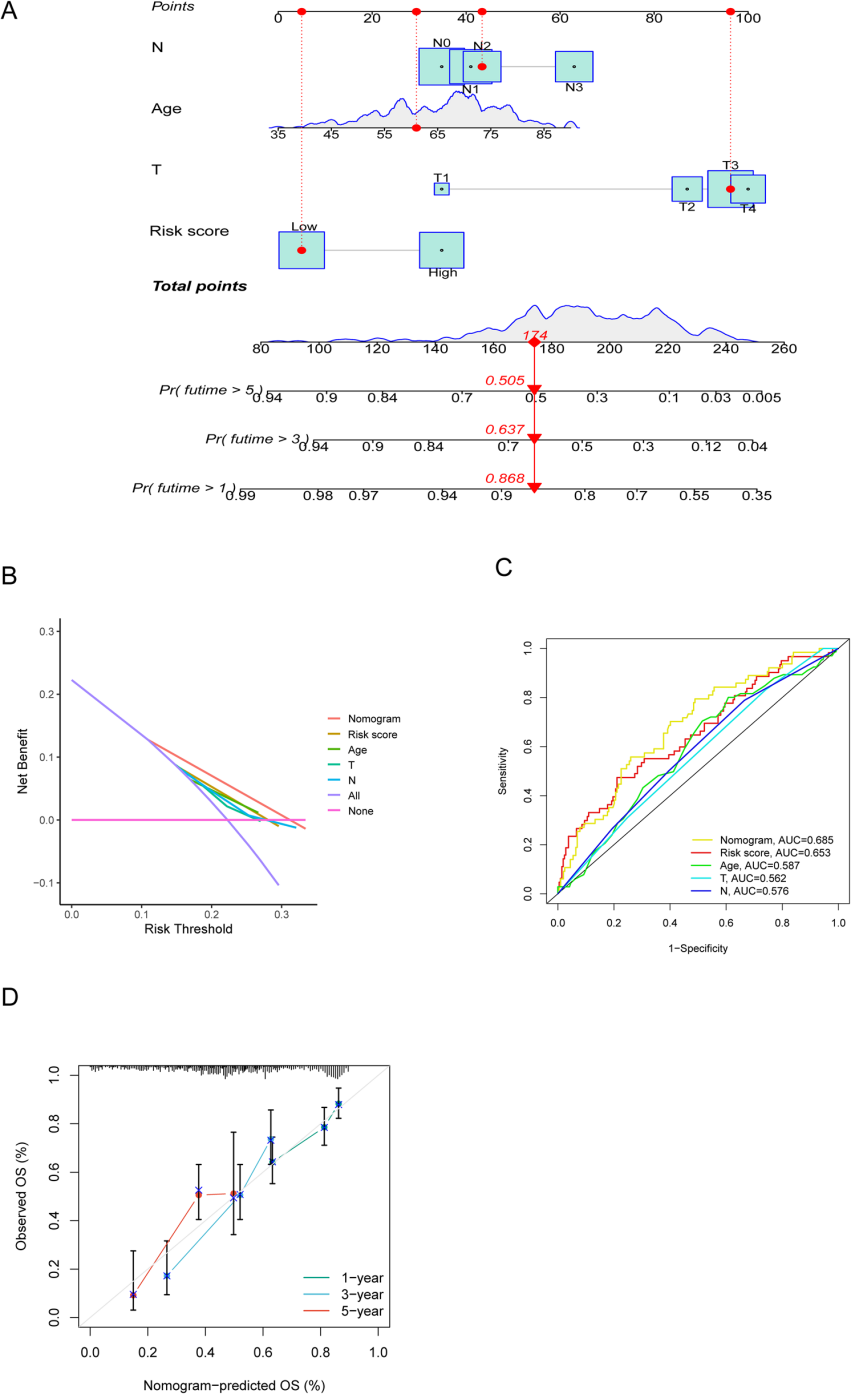
The construction and assessment of nomogram. (**A**) Nomogram integrating clinical factors and risk score for predicting 1-, 3-, and 5-year OS in TCGA-GC cohort (**B**) Decision curves of risk score, nomogram, and single clinical factors including T stage, N stage and age. (**C**) The time-dependent ROC curves of risk score, nomogram and single clinical factors including T stage, N stage and age. (**D**) The calibration curves for 1-, 3-, and 5-year OS. OS, overall survival.

To make the prognosis tool more convenient and quantitative, we integrated risk score with other clinical features including Age and TNM stage to establish a nomogram followed by a series of performance testing (Fig. 9A). The net benefit of nomogram was better than other clinical factors, a clinical value was observed as our expectations (Fig. 9B). The ROC curve analysis revealed that nomogram had an advantage over other single predictors. In addition, an excellent consistency with ideal model could be observed in the subsequent calibration plot of nomogram for OS predicting (Fig. 9C and D). Furthermore, to evaluate the prediction performance of the NRGsig for clinical applications in the TCGA-GC cohort, we compared our prognostic signature with other GC signatures reported in 2020 (Dai signature, Guan signature, Liu signature and Shao signature, respectively). We adopted similar risk score-estimated method described above towards these four signatures to generate risk score for samples from TCGA-GC cohort. The time-independent ROC curves illustrated that Liu signature, Shao signature and Guan signature exhibited lower AUC values for 1-, 3- and 5-year survival rates than NRGsig. The Dai signature presented similar AUC values with our signature (Fig. S8A–E). Similar to our signature, these four signatures could also predict the OS of GC patients except for Liu signature and shao signature (Fig. S8G–J). Moreover, the C-index of the NRGsig was the higher than other four signatures (Fig. S8K). NRGsig evidenced its advantage in long-term survival predicting and risk stratification compared with other four prognostic signatures.

### Comparison of the immune activity between subgroups

**Figure 10:**
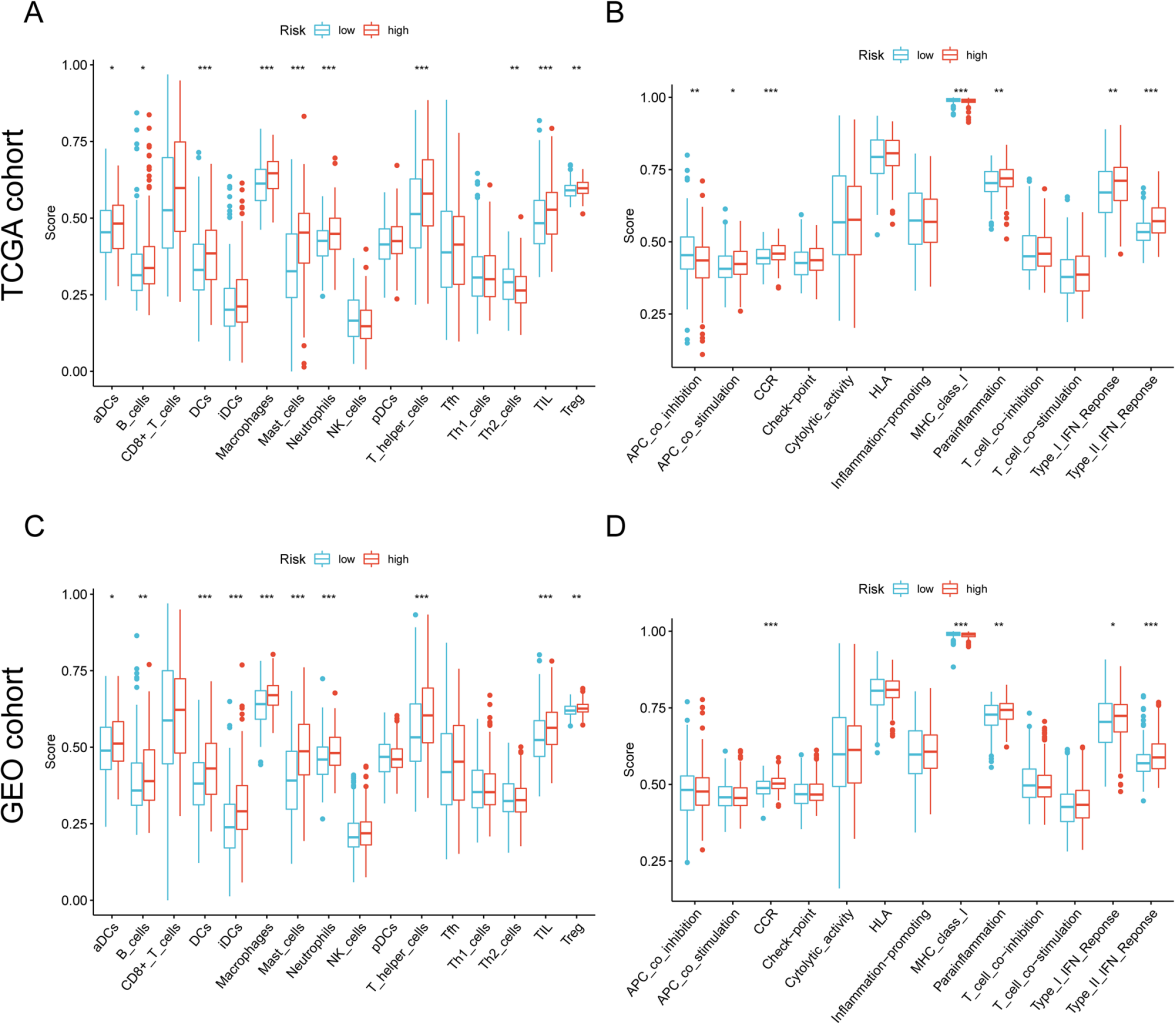
ssGSEA scores in the high- and low-risk group in the TCGA-GC and GSE84437 cohort. (**A**, **B**) TCGA cohort, (**C**, **D**) GSE84437 cohort. The scores of 16 immune cells (**A**, **C**) and 13 immune-related functions (**B**, **D**) are displayed in boxplots.

In line with our aim to increase the response to immunotherapy, we investigated the potential correlates between immune infiltration of tumors and NRGsig risk score. After calculating the infiltrating score of 16 immune cells and 13 immune-related pathways by using ssGSEA, we observed significantly increased antigen presenting function including aDCs, DCs and APC co-stimulation score in the high-risk group, while the activity of APC co-inhibition and MHC class I showed the opposite variation (all adjusted *P* < 0.05). Besides, contents of Treg cells, TIL cells and T helper cells were relatively higher in high-risk group, while the activity of Th2 cells had exactly the reverse results. Those results suggested significant difference in T cell regulation between the two subgroups. Moreover, CCR, mast cells, B cells, macrophages, neutrophils, parainflammation, type I IFN response and type II IFN response were observed to have increasing activities in samples from high-risk group (Fig. 10A and B). Similar observational results existed for in the GSE84437 cohort (Fig. 10C and D). Taken together, the findings of this study demonstrated that different risk groups have different immune landscape, which affected the prognosis of GC patients.

### Explorations of clinical applications for NRGsig

**Figure 11:**
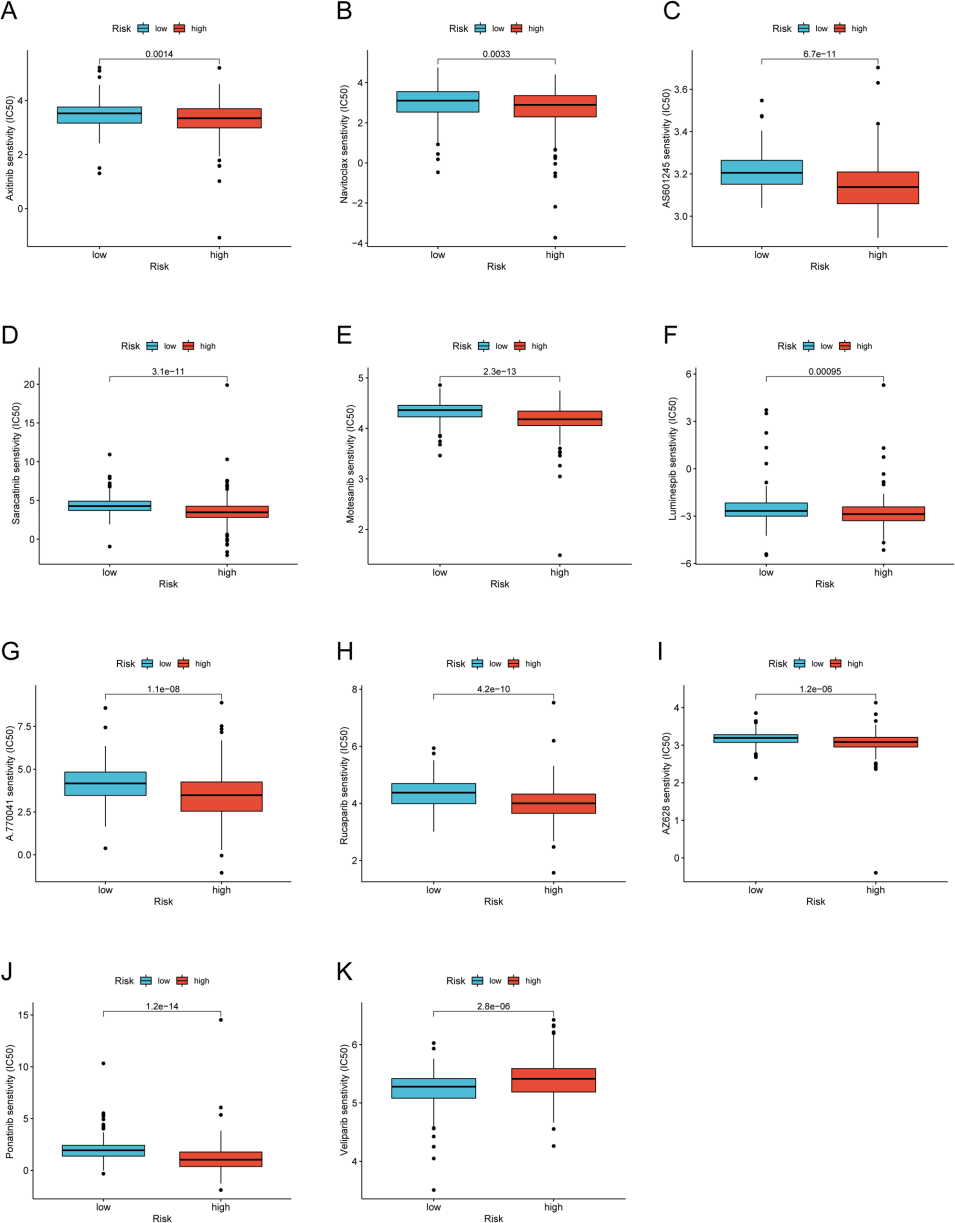
Drugs sensitivity analysis in patients from different risk score groups. The sensitivity to chemotherapeutic drugs was represented by the half-maximal inhibitory concentration (IC50) of chemotherapeutic drugs. (**A**–**K**) Comparisons of IC50 for chemotherapeutics drugs between two subgroups revealed that the high-risk group was more likely to benefit from the treatments (Kruskal–Wallis test, all *p* < 0.01).

We next explored potential expression changes of immune checkpoints between high- and low-risk groups. Results showed clear differences between the two patient groups, such as BTLA, CD86, CD200, CD27, and other immune checkpoints (Fig. S9). These results highlighted NRGsig as a therapeutic potential for combination strategies with immune checkpoint blockade (ICB) therapy in GC patients. Beyond ICB therapy, we also investigated sensitivity of chemotherapeutic and targeted therapeutics agents between high- and low-risk score groups in TCGA-GC cohort. Results indicated that IC50 toward eleven chemotherapeutics including A.770041, AS601245, AZ628, Axitinib, Luminespib, Navitoclax, Motesanib, Ponatinib, Rucaparib and Saracatinib, of samples in low-risk group were higher than those of high-risk group except for Veliparib (*P* < 0.05), suggesting that samples in low-risk group were more responsive to those medicine (Fig. 11A–K). As mentioned already, GSEA analysis revealed that a drug-resistant pathway like KEGG_BASE_EXCISION REPAIR was highly enriched in the low-risk score group, which could partially explain the above results. Drugs sensitivity analysis suggested that high-risk score patients might be more suitable for chemotherapy better response to chemotherapy.

**Figure 12:**
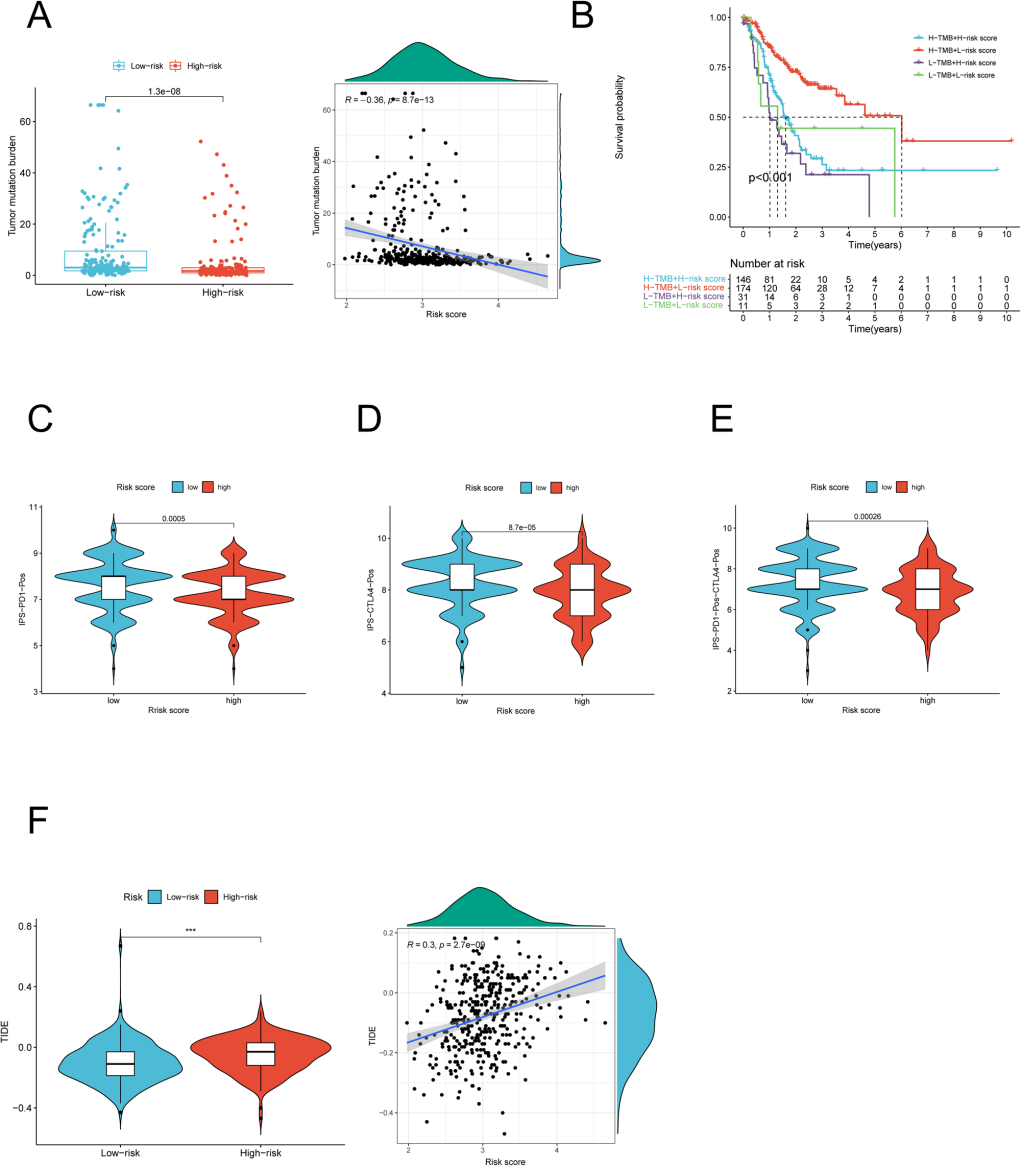
Correlation of risk score with TMB and predictive value of risk score for immunotherapy response. (**A**) TMB differences between the high- and low-risk score groups and the scatter plot depicted a positive correlation between risk score and TMB. (**B**) Kaplan–Meier curves for patients stratified by risk score and TMB in the TCGA-GC cohort. (**C**–**E**) Immunophenscore (IPS) between high- and low-risk score groups. Blue represents the low-score group and red the high-score group. The thick line within the violin plot represents the median value. The inner box between the top and bottom represents the interquartile range. (**C**) IPS score when PD-1 positive; (**D**) IPS score when CTLA4 positive; (**E**) IPS score when both PD-1 and CTLA4 positives. TMB, tumor mutation burden; IPS, Immunophenscore. (**F**) TIDE score differences between the high- and low-risk score groups and the scatter plot depicted a positive correlation between risk score and TIDE and lower risk score may be more likely to benefit from the immunotherapy (Spearman text, *p* < 0.001).

Evidence is growing that high TMB is a feature associated with response to immunotherapy in a variety of tumors, and high TMB levels lead to an increase in tumor neoantigens, which may trigger the immune system to attack the tumor^40,41^. Thus, we assessed the correlation of risk score with TMB in the TCGA-GC cohort. A negative relationship was observed between them, and the TMB score of the two risk groups were evaluated and significant disparity could be observed. The results illustrated that low-risk group patients had a significantly higher TMB than high-risk group (Fig. 12A). The combination of high TMB and low-risk score had the best OS in GC by Kaplan–Meier curves (Fig. 12B).

Furthermore, we explored the potential of risk score as predictor for immunotherapy response. We applied two mature algorithms, including IPS and TIDE, to predict the response of GC samples with different risk score to immunotherapy. The result evidenced that the IPS value for CTLA4 or PD1 therapy response was more sensitive in the low-risk group and suggested that the NRGsig has high potentiality for predicting CTLA4 and PD1 blockade therapy (Fig. 12C–E). On the other hand, the TIDE score was higher in the low-risk group and was also positively correlated with risk score, which indicated the lower risk score might benefit more from immunotherapy (Fig. 12F and G). Two distinct algorithms drew consistent results. The results above implied that NRGsig may effectively help predict the response to immunotherapy.

## Discussion

GC seriously threatens the health and life of Chinese people, with high morbidity, low early diagnosis rate and low survival rate^42^. Therefore, there is an urgent need to select specific relevant biomarkers for risk assessment to predict the prognosis of GC patients and facilitate the development of effective therapies for GC. Cell death is prevalent within tumors and has been proposed as a route for effective anti-cancer approach. Necroptosis is another common programmed cell death mode that activates and enhances antitumor immunity in cancer therapy, thus becoming a potentially practical cancer therapy^43,44^. However, few necroptosis-related prognostic signature have been developed for predicting personalized survival. Discovery of novel necroptosis-related prognostic signatures may provide important prognostic information and therapeutic targets for GC patients.

In this study, we first comprehensively evaluated the expression profile and genetic variation landscape of necroptosis-related genes in TCGA-GC patients. We found that 48 of 67 necroptosis genes were differentially expressed in cancer and normal tissues. At the genetic level, 147 of 433 patients were found to have undergone mutations with mutation frequencies ranging from 1 to 5%, with ATRX having the highest mutation of all necroptosis regulators. Next, we classified the GC patients in the meta-cohort according to the expression of DENRGs. The cluster C1 had a better survival advantage compared to the cluster C2. We also found that the levels of immune cell infiltration in the cluster C2, especially immune-suppressive cells (Tregs, Macrophages M2), were significantly higher than in the cluster C1, while the cluster C1 showed the opposite phenomenon with the immune-active cells (Tfh, activated T cell CD4 memory and Macrophages M1). In other words, the high expression of DENRGs in GC increased the high risk of tumor formation and led to the emergence of “cold tumors”^45^ (cluster C2), forming an immunosuppressive TME, weakening efficacy of cancer immunotherapy, causing poor clinical outcome. Our findings showed that the two necroptosis subtypes have distinct clinical prognostic outcomes and TME infiltration characteristics. Functional enrichment analysis revealed that DEGs mainly exerted immune-related functions and participated in tumor-related pathways, including extracellular matrix organization, extracellular matrix binding, ECM-receptor interaction, focal adhesion and TGF-beta signaling pathway.

To accurately predict the prognosis of individual GC patients, we constructed a prognostic gene signature based on these phenotype-related differential genes, which was named NRGsig. The Kaplan–Meier curve suggested that high-risk score patients tend to have worse outcomes. The PCA and t-SNE analysis demonstrated that the GC patients in the different risk groups were distributed in two directions. The time-dependent ROC curve demonstrated the NRGsig's had a good predictive potential. Furthermore, in univariate and multivariate Cox regression analysis, risk score was found to be an independent factor affecting the prognosis of GC patients. We identified differences in immune cell-related pathways by ssGSEA, which revealed that patients in two risk groups had different abundance of tumor-infiltrating immune cells and enriched immune-related pathway. The results above were reconfirmed by the independent GSE84437 cohort.

The AJCC TNM staging system, which known as the global standard for most cancer staging including GC, were generally used for assessment of tumor progression and prognosis prediction in clinical. It should be noted that patients with the same cancer stage often have disparate clinical course and varied clinical outcome. Recently, efforts have been made to assist and improve the AJCC staging system by integrating other additional characteristics^46–48^. There is urgent need for a clinical prognosis tool that is not only reliable and accurate but also practical and intuitive and it gives rise to nomogram, an mathematical scoring system^49^. By consider together several independent prognostic factors, nomogram works out robust predicting results of clinical outcome, including death or disease recurrence^50^. Therefore, we conducted a nomogram that could be used as a novel tool to quantify the prognosis of GC patients by combining risk score with other clinical variables and can aid in individualized therapy. The ROC curve, calibration plot and DCA curve all showed that the nomogram was a good prognostic tool. Results of the signature comparison analysis suggested that our signature has more advantages than other signatures.

In addition, we determined the drug sensitivity of different anticancer drugs in the treatment of patients with GC in distinct risk score groups. Based on IC50 values, Axitinib, Luminespib, Navitoclax, Motesanib, Ponatinib, Rucaparib and Saracatinib showed better responses in the high-risk score group. Screening chemotherapeutic drugs based on the molecular subtype of GC patients may allow more patients to benefit from individualized therapies. The above results indicated that NRGsig might be useful for guiding individualized treatments for GC patients. This preliminary result prompted us seek the relationship between the risk score and immunotherapy.

Targeted ICB therapy has been considered to be a promising way to treat cancer for some years^51^. However, only a minority of patients are sensitive to immune checkpoint inhibitors^52,53^. Currently, TMB serve as robust predictors of ICB treatment response in many malignancies, with high TMB generally reflecting better immunotherapy efficacy^54,55^. Our study revealed that the TMB score was different between two risk groups and significantly negatively correlated with risk score, indicating that the risk score might reflect the GC patients's response to immunotherapy to some extent. Previous studies have demonstrated that immune checkpoints can facilitate tumor cells to evade immune responses of the body^56,57^. We found that 22 of the 28 differentially expressed immune checkpoint genes were high expression in the high-risk score group. These results suggest that tumor cells in the high-risk score group evade immune attack by expressing immune checkpoint molecules, which in turn induce the formation of immunosuppressive microenvironment. This may partially explain potential reasons for bad prognosis in the high-risk score group patients. Therefore, the NRGsig may be used to predict immunotherapy response in GC. To further reconfirmed the response of GC samples with different risk score to immunotherapy, we applied two mature algorithms, including IPS and TIDE. IPS analysis and TIDE analysis were two methods most frequently applied in bioinformatic studies^58–62^. Two distinct algorithms drew consistent results that NRGsig might serve as an effective predictor for immunotherapy. Thus, a prognostic signature based on necroptosis may provide new insights into the prediction of immunotherapy outcome in GC.

However, we also recognize that there are two limitations to our study that cannot be ignored. First, the proposed prognostic signature in the present study was established and validated using retrospective data from public databases. Future prospective studies are required to verify its clinical utility; second, our research relies heavily on computational analysis, additional vivo and vitro experiments are important to verify these results above in the future.

## Conclusion

In summary, our study has established an accurate and effective prognostic signature to predict survival and immunotherapy response for GC patients. Moreover, we also established a novel nomogram which integrated the risk score and other clinical features and could help to develop individualized treatment plans based on the survival rates of individual patients.

## Supplementary Information

Supplementary Information.

## Data Availability

The raw data of this study are derived from the TCGA (https:// portal.gdc.cancer.gov/) and GEO (https://www.ncbi.nlm.nih.gov/geo/), which are publicly available databases.

## Abbreviations

GC: Gastric cancer
TME: Tumor microenvironment
NRGs: Necroptosis related genes
DENRGs: Differentially expressed NRGs
NRGsig: NRGs-based genetic signature
OS: Overall survival
TCGA: The cancer genome atlas
GEO: Gene expression omnibus
KEGG: Kyoto encyclopedia of genes and genomes
GO: Gene ontology
BP: Biological processes
MF: Molecular function
CC: Cellular components
PPI: Protein–protein interaction
PCA: Principal component analysis
t-SNE: T-distributed stochastic neighbor embedding
CNV: Copy number variations
DEGs: Differentially expressed genes
FDR: False discovery rate
ssGSEA: Single-sample gene set enrichment analysis
ROC: Receiver operating characteristic
DCA: Decision curve analysis
GSEA: Gene set enrichment analysis
TMB: Tumor mutation burden
IPS: Immunophenoscore
TCIA: The cancer immunome atlas
TIDE: Tumor immune dysfunction and exclusion
